# Can the intra-operative measurement of the diameter of the femoral head help surgeons to choose the best size of the acetabular cup?

**DOI:** 10.1007/s00264-022-05526-7

**Published:** 2022-08-11

**Authors:** Ernesto Muñoz-Mahamud, Clara Chimeno, Eduard Tornero, Alfonso Alías, Jenaro Ángel Fernández-Valencia, Andrés Combalia

**Affiliations:** 1 Servei de Cirurgia Ortopèdica i Traumatologia, Hospital Clínic de Barcelona, Universitat de Barcelona (UB), c. Villarroel, 170, 08036 Barcelona, Spain; 2 Departament de Cirurgia i Especialitats Medicoquirúrgiques, Facultat de Medicina i Ciències de la Salut, Universitat de Barcelona (UB), c. Casanova, 143, 08036 Barcelona, Spain; 3 Institut d’Investigacions Biomèdiques August Pi i Sunyer (IDIBAPS), c. Villarroel, 170, 08036 Barcelona, Spain; 4 Facultat de Medicina i Ciències de la Salut, Universitat de Barcelona (UB), c. Casanova, 143, 08036 Barcelona, Spain

**Keywords:** Intra-operative measurement, Femoral head, Acetabular cup, Hip template, Pre-operative template

## Abstract

**Purpose:**

We hypothesized that the intra-operative measurement of the femoral head may increase the accuracy of the acetabular cup size optimal selection in total hip arthroplasty (THA). The purpose of this clinical research was to analyze the correlation between the estimated cup size from intra-operative measurement of the femoral head and the pre-operative templated cup size.

**Methods:**

A prospective observational single-center study was conducted from June 2019 to January 2020 including primary THA (*n* = 100). All cases were pre-operatively templated. The measurement of the anterior–posterior diameter of the femoral head was routinely intra-operatively performed. Any definitive implanted cup was considered as “oversized” when the size was > 4 mm than the diameter of the native head.

**Results:**

The median (interquartile range) size of the implanted cup, pre-operative planned cup size, and diameter of the femoral head were measured 52 (50–54) mm, 50 (48–54) mm and 49 (45–51) mm, respectively. Pre-operative planned size cup accurately predicted the implanted cup or differed in only one size (2 mm) in 77 (78%) cases. Otherwise, intra-operative femoral head measurement method accurately predicted the implanted or differed in only one size (2 mm) in 51 (87%) cases (*p* = 0.097).

**Conclusion:**

The intra-operative femoral head measurement is a simple and reliable tool to help the surgeons choose the best size of the acetabular cup and is as reliable as the pre-operative templating in order to avoid cup oversizing in THA. Utmost caution is warranted whenever the cup reamer is > 4 mm than the anterior–posterior diameter of the native head.

**Supplementary Information:**

The online version contains supplementary material available at 10.1007/s00264-022-05526-7.

## Introduction

Total hip arthroplasty (THA) is an exceptionally successful and cost-effective surgical procedure [1, 2]. In order to obtain reproducible results, pre-operative planning has emerged as a mandatory routine and has become a cornerstone step so as to achieve remarkable outcomes [3, 4]. This planning includes physical examination and templating using x-ray calibration tools [5] which help in the selection of proper component sizes and in the assessment of leg–length discrepancy [6].

Odri et al. reported significant higher post-operative pain among those cases in which the diameter difference between the original femoral head and the implanted socket was superior to 6 mm [7]. Accordingly, Ben Lulu et al. suggested a real-time tool for the accuracy of acetabular size selection, consisting on an intra-operative measurement of the femoral head and considering a cut-off point of 4 mm as a monitoring indicator [8].

We hypothesize that this simple tool may have the same validity and accuracy as the pre-operative digital templating in order to estimate the size of the definitive implanted cup. The objective of the current study was to analyze the correlation between the estimated cup size from intra-operative measurement of the femoral head and the pre-operative templated cup size.

## Materials and methods

This was an observational single-centre study. From June 2019 to January 2020, all patients admitted at our hospital for elective THA were prospectively registered in a database and retrospectively reviewed. We excluded those patients undergoing THA with no pre-operative template, patients in which the femoral head was non-measurable for any reason, and patients in whom data collection was incorrect or inadequate due to clerical errors.

All operations were performed by at least one of the joint reconstruction surgeons following the usual technique. Data regarding demographics, body mass index (BMI), comorbidities, indication for THA, hip approach, anterior–posterior diameter of the femoral head, templated socket size and implanted cup outer diameter, and type of cup was recorded. The type of cup implanted was R3™ (Smith & Nephew, Memphis, TN), G7™ (Zimmer Biomet, Warsaw, IN), Trident ™ (Stryker Orthopaedics, Mahwah, NJ), or X3 RimFit ™ (Stryker Orthopaedics, Mahwah, NJ). All mentioned cup models have various sizes that differ in exactly 2 mm from the immediate, and only even sizes are available. This fact was taken into account in the estimation from the different methods of the definitive implanted size cup.

### Estimated cup size method from pre-operative templating x-ray calibration tools (pre-operative planned cup)

**Fig. 1:**
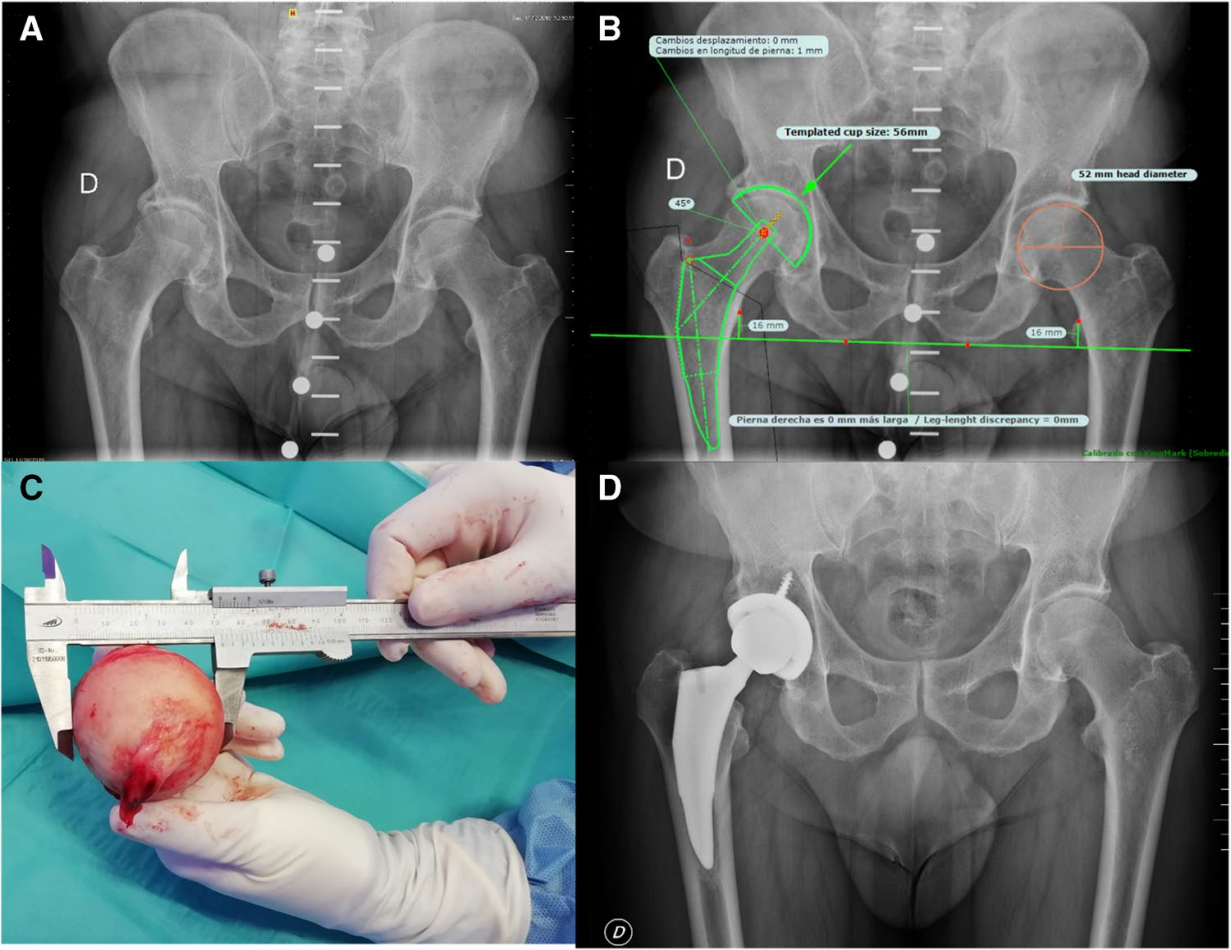
**A** Digital pre-operative radiograph showing the anterior–posterior projection of the pelvis with a dual calibration marker ball system (KingMark™). **B** Pre-operative template using the using the TraumaCad™ software in which a 56 mm diameter head was estimated. **C** Intra-operative measurement of the anterior–posterior diameter of the femoral head using a Vernier caliper device. In this example, a 52 mm diameter head was measured. **D** Post-operative radiograph after the implantation of a definitive 56 mm diameter cup

All digital preoperatively radiographs of the pelvis were taken in an anterior–posterior orientation. Patients were positioned supine with both hip joints rotated inward approximately 10–15º. A dual calibration marker ball system (KingMark™) was routinely used as a reference for determining the individual magnification factor [9] (Fig. 1A). Digital templating was performed as described by Bono et al. [10] using the TraumaCad™ (BrainLab, Chicago, IL, USA) (Fig. 1B). The increments in size of all cups are 2 mm (external diameter of the cup); thus, only even sizes from all models are commercially available. The implant type was determined pre-operatively based on the patient’s individual anatomy, age, and weight.

### Estimated cup size method from intra-operative femoral head measurement

The measurement of the anterior–posterior diameter of the femoral head was intraoperatively performed using a Vernier caliper device (Fig. 1C). Considering a cut-off point of 4 mm as a monitoring indicator [8] and the fact that only even sizes from all models were commercially available, the estimated cup size was calculated as the immediate available even-sized cup ≤ 4 mm larger than de diameter of the femoral head.

### Definitive implanted cup size selection method

In all cases, a progressive acetabular reaming was performed preferably not exceeding 4 mm the diameter of the measured native femoral head. An acetabular cup trial was impacted so as to test the press-fit**.** The definitive implanted cup size was selected according to both the subjective surgeon’s sensation after using the cup trial and the preoperatively templated x-ray. After the implantation of the definitive cup (and a stem trial) and reduction, intraoperative x-rays were performed in all cases in order to accurately assess well positioning of the implants and reproducibility of the pre-operatively templated x-ray. Post-operative x-rays were performed in all cases (Fig. 1D).

### Oversized cup definition

For analysis purposes, any definitive implanted cup was considered as “oversized” when (a) the size was > 4 mm than the anterior–posterior diameter of the native head or (b) the implanted cup was larger than the pre-operative planned cup.

### Statistical analysis

Continuous variables were expressed as mean or median and standard deviation (SD) or interquartile range (IQR) and were compared using the Student’s *t* test or the Mann–Whitney *U* test according to the Kolmogorov–Smirnov test of normality. Qualitative variables were described by absolute frequencies and percentages and were compared using the χ^2^ test or Fisher’s exact test when necessary. Correlation curves between continuous variables were estimated by testing linear equation models. Lin’s concordance correlation coefficient [11] was calculated to assess the correlation between implanted and predicted size cup. The degree of agreement was assessed according to McBride’s strength of agreement criteria [12] for discrete quantitative variables (< 0.65, poor; 0.65–0.80, moderate; 0.80–0.90, substantial; > 0.90, almost perfect). Analyses were performed using the SPSS® v. 20.0 statistical package (SPSS, Inc. Chicago, IL, USA). Two-sided *p* ≤ 0.05 was considered statistically significant. Institutional review board approval was obtained before the beginning of the study (Register number: HCB/2020/0494).

## Results

A total of 100 patients were included in the study. The mean age of the cohort was 65.1 (SD, 13.3) years. Fifty-six (56%) patients were male, and mean BMI was 27.7 (SD, 4.7) Kg/m^2^. Indications for surgery were osteoarthritis (84 cases, 84%), ischemic necrosis of the femoral head (10 cases, 10%), femoral neck fracture (5 cases, 5%), and malignancy in 1 (1%) case. Hip surgical approach was anterolateral in 70 patients, direct anterior in 18, and posterolateral approach in 12 patients. Hip prosthesis was non-cemented in 98 (98%) cases, and the type of cup implanted was R3™ (Smith & Nephew, Memphis, TN, USA) in 45 cases (45%), G7 ™ (Zimmer Biomet, Warsaw, IN, USA) in 39 cases (39%), and Trident ™ (Stryker, Kalamazoo, MI, USA) in 13 cases (13%). There were two cases (2%) of cemented cups, and the type was X3 RimFit ™ (Stryker, Kalamazoo, MI, USA).

**Fig. 2:**
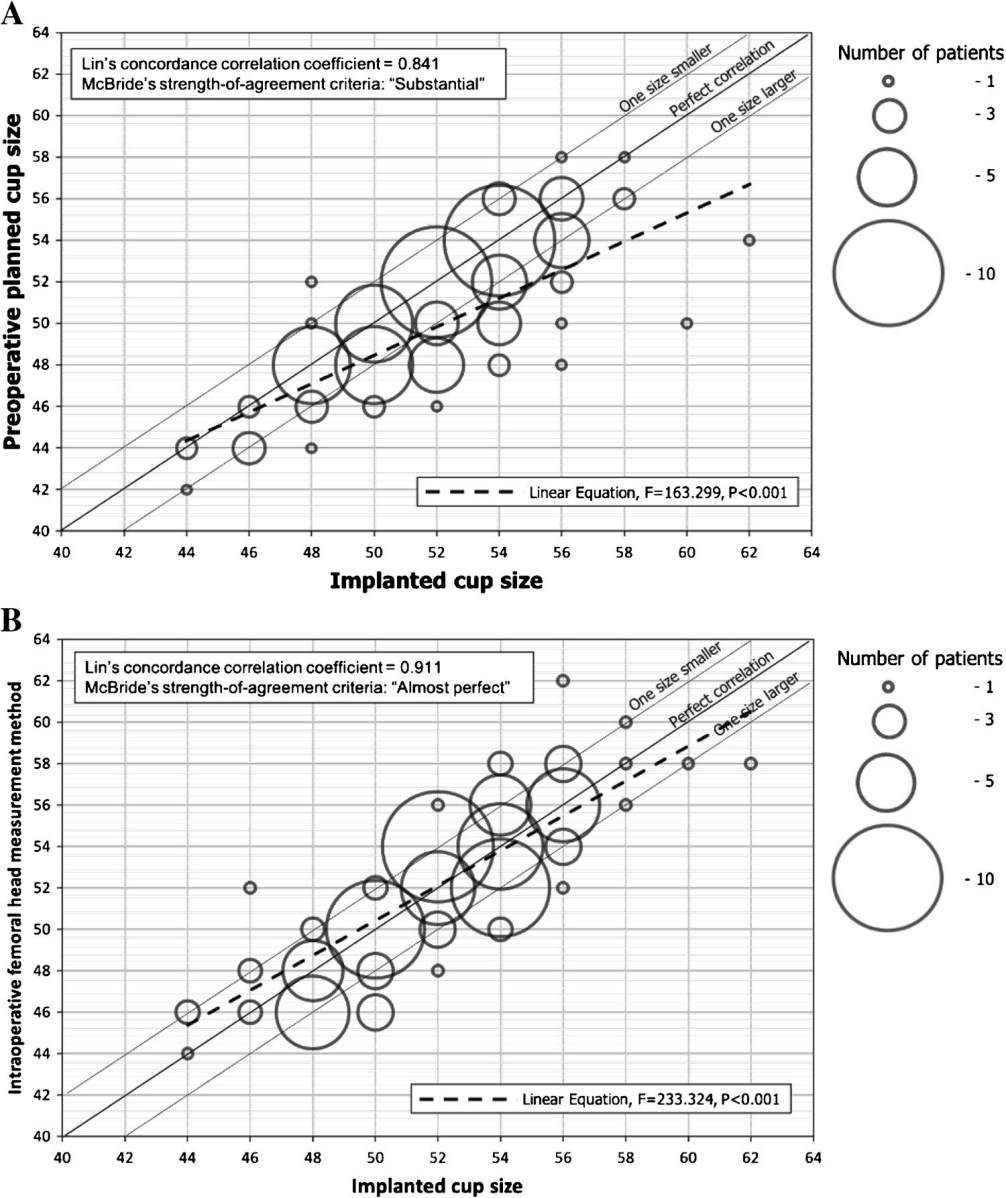
**A** Linear correlation between definitive implanted cup size and pre-operative planned cup size. **B** Linear correlation between definitive implanted cup size and intra-operative femoral head measurement method (measurements are expressed in mm). The bubble diameter indicates the number of patients with that agreement

The median (IQR) size of the implanted cup was 52 (50–54) mm, whereas the median (IQR) pre-operative planned cup was 50 (48–54) mm, and the median (IQR) size of the femoral head (measured with Vernier caliper during surgery) was 49 (45–51) mm. Figure 2 shows correlation between implanted cup and both the pre-operative planned cup and the intra-operative femoral head measurement method (as described in methods section). Lin’s concordance correlation coefficient between implanted cup and preo-perative planned cup was 0.841 (95%CI = 0.601–0.920); therefore, the strength of agreement was defined as “substantial.” The correlation coefficient between implanted cup and intra-operative femoral head measurement method was 0.911 (0.868–0.940), and the strength of agreement was categorized as “almost perfect.” Pre-operative planned size cup accurately predicted the implanted cup or differed in only one size (2 mm) in 77 (78%) cases (Table 1). Otherwise, intra-operative femoral head measurement method accurately predicted the implanted or differed in only one size (2 mm) in 51 (87%) cases (*p* = 0.097).

**Table 1:** Percentage of accurately prediction or discrepancy of both the pre-operative planned cup diameter and the intra-operative femoral head measurement compared to the definitively implanted cup size. Comparison of proportions (*z*-test)

	Pre-operative templated cup size	Intra-operative femoral head measurement	*p* value
Percentage of accurately prediction	43%	36%	0.31
Percentage of discrepancy ≤ 1 size	35%	51%	0.02
Percentage of discrepancy > 1 size	22%	13%	0.10

## Discussion

Pre-operative planning in THA has become a well-recognized and widely accepted method to improve implant sizing, determine offset, and reduce leg length discrepancy [13]. In fact, its routinely application has demonstrated to optimize the femoroacetabular offset adjustment by determining component sizes [14]. Unfortunately, pre-operative template might not be always feasible, for instance, in the absence of sizing marker or malposition of the patient during the radiographs. Another usual situation in which the pre-operative templating may be difficult is related to hip fracture cases, in which non-standardized x-rays are common and thus sizing marker may be unavailable. Finally, it should be taken into account that not all centres are provided with a proper software to digitally template. In fact, pre-operative planification has been classically done with hand-sketched plans [3].

Crosswell S. et al. [15] devised an easy method so as to help predicting femoral head size, therefore allowing surgeons to safely proceed with surgery when implant stocks are limited and to potentially improve theater efficiency. The authors described a simple and reproducible method in which the maximum diameter of the contralateral femoral head using a digital software was measured. However, this study was addressed to pre-operative template hip hemiarthroplasties, so further studies should be performed in order to assess the real usefulness of this technique regarding the use of THA for hip fracture. Other authors have used templating programs to measure the widest point on the femoral head. However, a contralateral hip joint configuration may vary individually and differ from the affected limb. According to the presented results, the intra-operative measurement of the femoral head provides high reliability to choose the cup size. This method poses a valuable aid in cases in which the pre-operative planning is not available.

A difference higher than 6 mm between the native femoral head and the implanted cup has been depicted as a potentially preventable cause for post-operative pain after THA [7]. A simple and reproducible real-time method so as to improve the accuracy of acetabular size selection was described by Ben Lulu et al. [8], consisting an intra-operative measurement of the resected native head and considering a cut-off point of 4 mm as a monitoring indicator. This basic yet appealing method consists of an unexpensive widely available tool that should be considered as routine whenever an acetabular cup is implanted. In the present series, the intra-operative femoral head measurement method provided greater accuracy (87%) compared to the standard pre-operative templating (78%). The linear correlation demonstrates that the intra-operative femoral head measurement is at least as reliable as the pre-operative templating. This reproducible and easy tool appears to be useful not only as a double check to confirm our pre-operatively estimated cup size, but also as an aid in those cases in which the pre-operative template is unavailable for any reason.

The present study features some inherent limitations. The first limitation is that different cup models were used during the study period, which adds heterogenicity. However, all used models have equal even sizes which differ in 2 mm from the immediate. Secondly, the number of cases is limited and may have influenced the results. Finally, there might be some challenging cases in which the femoral head is totally deformed and cannot be reliably measured (for instance, some severe avascular necrosis and high-grade dysplasia cases). In those cases, we certainly encourage relying on the pre-operative template, considering the contralateral femoral head (if not deformed too) as a guide. All these limitations may affect the extent to which our findings can be generalized beyond the specific cases studied. There are some strengths that need to be highlighted. Firstly, because of its prospective nature, biases have been reduced to the maximum. Secondly, all operations were performed by the same specialized surgeons of the hip unit using the same surgical protocols. In all, it can be assumed that future larger prospective randomized studies are required to corroborate our findings.

In conclusion, the intra-operative femoral head measurement is as reliable as the pre-operative templating in order to avoid cup oversizing in THA. This simple and reproducible tool has proved to be useful to confirm the pre-operatively predicted cup size; thus, its application should be considered routinely, even in those cases in which the pre-operative template is available. In all, the intra-operative femoral head measurement entails an unexpensive widely available tool that should be considered as routine in THA.

## Supplementary Information

Below is the link to the electronic supplementary material.

Supplementary file1 (SAV 6 KB)

## Data Availability

All research data has been uploaded as an anonymized dataset in Supplementary Information.

## References

[1] Bourne RB, Maloney WJ, Wright JG (2004). An AOA critical issue. The outcome of the outcomes movement. J Bone Joint Surg Am.

[2] Chang RW, Pellisier JM, Hazen GB (1996). A cost-effectiveness analysis of total hip arthroplasty for osteoarthritis of the hip. JAMA.

[3] Eggli S, Pisan M, Muller ME (1998). The value of preoperative planning for total hip arthroplasty. J Bone Joint Surg (Br).

[4] Garvin K (2005) Primary total hip arthroplasty: preoperative planning and templating. In: Lieberman J, Berry D (eds) Advanced reconstruction hip. Rosemont (III), edited by the American Academy of Orthopaedic Surgeons, p 41

[5] Gallart X, Daccach JJ, Fernández-Valencia JÁ, García S, Bori G, Rios J, Riba J (2012). Study of the consistency of a system for preoperative planning digital in total arthroplasty of the hip. Rev Esp Cir Ortop Traumatol.

[6] Maloney WJ, Keeney JA (2004). Leg length discrepancy after total hip arthroplasty. J Arthroplasty.

[7] Odri GA, Padiolleau GB, Gouin FT (2014). Oversized cups as a major risk factor of postoperative pain after total hip arthroplasty. J Arthroplasty.

[8] Ben Lulu O, Rubin G, Krasnyansky S, Elbaz A, Segal G, Rozen N (2015). Measuring the femoral head size–an additional real-time intraoperative monitoring tool for the accuracy of the preoperative process and implant selection. J Arthroplasty.

[9] King (2009). A novel method of accurately calculating the radiological magnification of the hip. J Bone Joint Surg Br.

[10] Bono JV (2004). Digital templating in total hip arthroplasty. J Bone Joint Surg Am.

[11] Lin LI (1989). A concordance correlation coefficient to evaluate reproducibility. Biometrics.

[12] McBride GB (2005) A proposal for strength-of-agreement criteria for Lin's Concordance Correlation Coefficient. NIWA Client Report HAM May 2005-062, NIWA Project MOH05201

[13] Valle AGD, Slullitel G, Piccaluga F, Salvati EA (2005). The precision and usefulness of preoperative planning for cemented and hybrid primary total hip arthroplasty. J Arthroplasty.

[14] Stigler SK, Müller FJ, Pfaud S, Zellner M, Füchtmeier B (2017). Digital templating in total hip arthroplasty: additional anteroposterior hip view increases the accuracy. World J Orthop.

[15] Crosswell S, Akehurst H, Ramiah R, Navadgi B (2019). Preoperative sizing of hip hemiarthroplasties to accurately estimate head size from non-standardised pelvic radiographs: can it be done?. Injury.

